# MEANING IN LIFE AND REASONS FOR LIVING IN OLDER ADULTS WITH SUICIDAL IDEATION: AN INTEGRATIVE REVIEW

**DOI:** 10.1093/geroni/igz038.996

**Published:** 2019-11-08

**Authors:** Atami Sagna

**Affiliations:** The University of Texas at Austin, Austin, Texas, United States

## Abstract

Suicide has ranked as the 10th leading cause of death for all ages in the United States. Although Healthy People 2020’s target is to reduce suicide rates by 10.2 per 100,000 by 2020, it remains that suicide rates continue to increase, with suicide in older adults contributing substantially to this rise. Older adults have a higher risk for suicide, yet research in the area on positive psychological factors such as meaning in life and reasons for living is lacking. The purpose of this review is to investigate the associations among meaning in life, reasons for living and suicidal ideation in older adults (55+ years). Based on PRISMA guidelines, the PubMed, PsycINFO, PsycARTICLES, and CINAHL databases were systematically searched for relevant publications without date restrictions. Nine studies, qualitative and quantitative, are included in the review, showing a relationship among meaning in life, reasons for living and suicidal ideation in older adults. All the studies found that meaning in life and reasons for living were negatively associated with suicidal ideation in older adults. The findings of this review highlight the importance of including positive psychological factors in assessing suicide risk in older adults and in planning preventative measures and services for this high-risk group.

